# Reduced chemokine C‐C motif ligand 1 expression may negatively regulate colorectal cancer progression at liver metastatic sites

**DOI:** 10.1111/jcmm.18193

**Published:** 2024-03-20

**Authors:** Miku Iwata, Ryuma Haraguchi, Riko Kitazawa, Chihiro Ito, Kohei Ogawa, Yasutsugu Takada, Sohei Kitazawa

**Affiliations:** ^1^ Department of Molecular Pathology Ehime University Graduate School of Medicine Toon City Ehime Japan; ^2^ Department of Hepato‐Biliary‐Pancreatic and Breast Surgery Ehime University Graduate School of Medicine Toon City Ehime Japan; ^3^ Division of Diagnostic Pathology Ehime University Hospital Toon City Ehime Japan

**Keywords:** CCL1, CCR8, CRC, CRLM, TAM

## Abstract

Colorectal cancer (CRC) liver metastasis, albeit a stage‐IV disease, is completely curable by surgical resection in selected patients. In addressing the molecular basics of this phenomenon, differentially expressed genes at primary and liver metastatic sites were screened by RNA sequencing with the use of paraffin‐embedded surgical specimens. Chemokine C‐C motif ligand 1 (CCL1), a chemotactic factor for a ligand of the chemokine C‐C motif receptor 8 (CCR8), was isolated as one of the differentially expressed genes. Histological analysis revealed that the number of CCL1‐positive cells, mainly tumour associated macrophages (TAMs) located in the stroma of CRC, decreased significantly at liver metastatic sites, while the expression level of CCR8 on CRC remained unchanged. To explore the biological significance of the CCL1‐CCR8 axis in CRC, CCR8‐positive CRC cell line Colo320DM was used to assess the effect of the CCL1‐CCR8 axis on major signalling pathways, epithelial mesenchymal transition induction and cell motility. Upon stimulation of recombinant CCL1 (rCCL1), phosphorylation of AKT was observed in Colo320DM cells; on the other hand, the corresponding significant increase in MMP‐2 levels demonstrated by RT‐qPCR was nullified by siRNA (siCCR8). In the scratch test, rCCL1 treatment significantly increased the motility of Colo320DM cells, which was similarly nullified by siCCR8. Thus, the activation of the CCL1‐CCR8 axis is a positive regulator of CRC tumour progression. Reduced CCL1 expression of TAMs at liver metastatic sites may partly explain the unique slow tumour progression of CRC, thus providing for a grace period for radical resection of metastatic lesions.

## 1 INTRODUCTION

Colorectal cancer (CRC) is the third most common cancer worldwide and was the second leading cause of cancer‐associated mortality in 2020.^1^ Approximately 50% of patients with CRC are diagnosed with synchronous or metachronous liver metastasis,^2^ and the number of patients with colorectal liver metastasis (CRLM) eligible for surgical resection has increased because of advances in surgery and chemotherapy. Surgical resection of liver metastatic sites does improve CRC patient prognosis. A Japanese multicenter analysis has demonstrated 5‐year overall survival (OS) rate of 47.7% for patients after hepatectomy for CRLM, whereas that of patients after hepatectomy for liver metastasis of gastric and pancreatic cancer was 32% and 31%, respectively.^3^, ^4^, ^5^ Furthermore, in even most cases of recurrent CRLM that are often limited to intrahepatic metastasis, repeated hepatectomy is also effective.^6^ Thus, surgical treatment is currently preferentially chosen for the selected patients with CRC diagnosed with CRLM.

Tumour associated macrophages (TAMs) and cancer associated fibroblasts (CAFs) are major factors that constitute the tumour microenvironment (TME) and modulate tumour progression and metastasis.^7^, ^8^ The infiltration of TAMs into most solid tumours has been correlated with poor prognosis, and results in the immunosuppressive microenvironment by secreting various chemokines and immunosuppressive factors.^11^, ^12^, ^13^ Regardless of some contradictory reports,^9^, ^10^ TAMs in CRC have also been described as one of the factors promoting tumour progression. Here, assuming that the altered character of TAMs decelerates CRC progression at liver metastatic sites, the differentially expressed genes compared with those at the primary site were isolated by RNA sequencing with the use of total RNA extracted from formalin‐fixed, paraffin‐embedded (FFPE) tissues by trimming the tumour site of CRC and CRLM.

## 2 MATERIALS AND METHODS

### 2.1 Patients and tissue samples

The samples archived at Ehime University (Toon, Ehime) were used in this study. Comprehensive written consent forms were obtained from the patients at admission and for conducting all relevant experiments.

Sixteen patients who had undergone surgical treatment for CRC and CRLM with no other distant metastasis at Ehime University Hospital between 2016 and 2021 were included in this study. Some patients underwent repeated hepatectomy for recurrent CRLM, two died of systematic metastasis 3 years after surgery and 14 patients are still alive.

### 2.2 RNA extraction and sequencing

Total RNA was extracted from FFPE tissue with the use of NucleoSpin® total RNA FFPE (Macherey‐Nagel, Duren, Germany) by following the manufacturer's protocols. The extracted RNA was treated with DNaseI (Macherey‐Nagel, Duren, Germany) to remove DNA. Total RNAs concentration and integrity were assessed with a NanoVue Plus™ Spectrophotometer (GE Healthcare, Chicago, Illinois, USA) and an RNA 6000 Nano Kit and Agilent 2100 BioAnalyzer (Agilent Technologies, Santa Clara, CA, USA), respectively. Library preparation, construction and sequencing were carried out at Advanced Research Support Center of Ehime University. A total of 100 ng RNA per sample was used for library preparation without fragmentation. Libraries were qualified with an Agilent High Sensitivity DNA Kit and Agilent 2100 BioAnalyzer (Agilent Technologies, Santa Clara, CA, USA), and quantified with a Kapa Library Quantification Kit (NIPPON Genetics, Tokyo, Japan). Finally, the purified libraries were pair‐end sequenced on the Illumina MiSeq system (Illumina, San Diego, CA, USA). Sequencing raw data were trimmed with Trim Galore! software and mapped to the hg38 reference genome with Hisat2 software. The RNA expression profiles were determined based on Transcripts Per Kilobase Million (TPM) normalization. The sequencing coverage and quality statistics of each sample are summarized in Table S1.

### 2.3 RNA scope® in situ hybridization

FFPE tissues were used for the RNA® scope assay. RNA scope® in situ hybridization was implemented with the RNA® scope 2.5 HD Assay–BROWN assay (Advanced Cell Diagnostics, Newark, CA, USA) according to the manufacturer's protocols. The target probe used was Hs‐CCL1 (cat.468541) and the positive control probe used was Hs‐PPIB (cat.313901).

### 2.4 Immunohistochemistry

The specimens were routinely fixed in 10% formalin and embedded in paraffin wax; 5‐μm slices were then deparaffinized, dehydrated and used for heat‐induced antigen retrieval with Tris‐EDTA buffer. The FFPE sections were stained with chemokine C‐C motif ligand 1 (CCL1) antibody (1:50; cat. no. HPA049861; Sigma‐Aldrich, St. Louis, MO, USA) and chemokine C‐C motif receptor 8 (CCR8) antibody (1:100; cat. no. NBP2‐15768; Novus Biologicals, Centennial, CO, USA) and incubated at 4°C overnight. HRP‐conjugated anti‐rabbit IgG (1:100; cat. no. K4003; DAKO, Agilent technologies, Santa Clara, CA, USA) was used as the secondary antibody at room temperature for 1 hour. Finally, colour was detected with an ImmPACT® DAB Peroxidase Substrate Kit (cat. no. SK4105, Vector Laboratories, Burlingame, CA, USA) according to the manufacturer's protocols, and the slides were counterstained with haematoxylin. Negative controls were tested for CCL1 and CCR8 staining with isotype control antibody rabbit immunoglobulin.

### 2.5 Double immunofluorescence staining

FFPE tissue sections were treated with rabbit polyclonal anti‐CCL1 antibody (1:50; cat. no. HPA049861; Sigma‐Aldrich, St. Louis, MO, USA) and mouse monoclonal recombinant anti‐CD68 (1:100; cat. no. M0876; DAKO, Agilent technologies, Santa Clara, CA, USA) for the detection of CCL1 and CD68 expression, respectively. Alexa Fluor 488‐ and 568‐coupled secondary antibodies (1:100; cat. no. A‐11008, A‐11004; Thermo Fisher Scientific, Walham, MA, USA) were also used for the detection. Images were acquired through a BIOREVO BZ‐9000 microscope (KEYENCE, Osaka, Japan).

### 2.6 Cell culture and reagents

Human colon carcinoma cell lines Lovo (RRID:CVCL_0399), HT‐29 (RRID:CVCL_0320), SW620 (RRID:CVCL_0547), WiDr (RRID:CVCL_2760), HCT116 (RRID:CVCL_0291), DLD‐1 (RRID:CVCL_0248) and Colo320DM (RRID:CVCL_0219) were obtained from the Japanese Collection of Research Bioresources (JCRB) Cell Bank (Osaka, Japan). WiDr (RRID: CVCL_2760) is an HT‐29 (RRID: CVCL_0320) derivative cell line. The cells were maintained in RPMI 1640 medium (Wako, Osaka, Japan) supplemented with 10% fetal bovine serum (Sigma‐Aldrich, USA, St. Louis, MO, USA) and 1% Antibiotic‐Antimycotic (Gibco, Thermo Fisher Scientific, Walham, MA, USA) at 37°C in a humidified incubator containing 5% CO_2_. All cell lines had been authenticated using short tandem repeat (STR) profiling within the last 3 years, and all experiments had been done with mycoplasma‐free cells. Recombinant Human CCL1 Protein (rCCL1) was purchased from R&D Systems (cat. 272‐I‐010, Minneapolis, NE, USA). MK‐2206 dihydrochloride, an Akt inhibitor, was purchased from MedChemExpress (cat. HY‐10358, Monmouth Junction, NJ, USA).

### 2.7 RNA extraction and reverse transcription‐quantitative PCR (RT‐qPCR) analysis

Total RNA (1 μg) extracted from Colo320DM cells with an RNeasy Mini Kit (Qiagen, Hilden, Germany) was reverse transcribed by using High‐Capacity cDNA Reverse Transcriptase (Applied Biosystems, Walham, MA, USA) according to the manufacturer's protocols. RT‐qPCR was carried out with cDNA by using TaqMan® Universal Master Mix (Thermo Fisher Scientific, Walham, MA, USA) in the ABI PRISM® 7500 real‐time PCR system (Applied Biosystems, Walham, MA, USA). The qPCR reaction was carried out at 50°C for 2 min, followed by 40 cycles at 95°C for 10 min and at 60°C for 1 min. The following TaqMan® Gene Expression assays (Thermo Fisher Scientific, Walham, MA, USA) were used to analyse the expression of each gene: CCR8 (Hs 00174764_m1), GAPDH (Hs 00266705_g1), CDH1 (Hs 01023894_m1), MMP‐2 (Hs 01548727_m1), MMP‐9(Hs 00957562_m1) and VEGFA (Hs 00900055_m1). To evaluate CCR8 mRNA expression in CRC cell lines, RT‐qPCR was implemented with cDNA synthesized with the THUNDERBIRD® SYBR® qPCR mix (Toyobo, Osaka, Japan) and the gene‐specific primer sets (Forward CCR8 primer sequences: 5′‐CTGCACTCACATGAGGATACAG‐3′, Reverse CCR8 primer sequences: 5′‐CACTGTTGTCACACTGAGGTC‐3′) were used according to the manufacturer's protocols. Relative expression levels of each gene were evaluated with the standard curve method and normalized to internal control GAPDH.

### 2.8 Western Blot analysis

Total proteins were extracted on ice for 30 min from Colo320DM cells using RIPA lysis buffer containing a 1% protease inhibitor and a 1% phosphatase inhibitor cocktail (Nacalai Tesque, Kyoto, Japan). Total protein concentration (5 μg/lane) was measured with a Pierce bicinchoninic acid protein assay kit (Thermo Fisher Scientific, Walham, MA, USA), separated by 5%–20% SDS‐PAGE, transferred onto a PVDF membrane (Merck Millipore, Burlington, Germany), blocked with 1% bovine serum albumin (Wako, Osaka, Japan) at room temperature for 30 min and incubated with a primary antibody overnight at 4°C. The following primary antibodies were used: Rabbit antibody against AKT (1:1000; cat. no. 9272; Cell Signaling Technology, Danvers, MA, USA), rabbit antibody against phosphorylated AKT (Ser473: 1:1000; cat. no. 4060; Cell Signaling Technology, Inc.), mouse antibody against p44/42 Erk1/2 (1:500; cat. no. 4696; Cell Signaling Technology, Danvers, MA, USA), rabbit antibody against phosphorylated p44/42 Erk1/2 (1:1000; cat. no. 4370; Cell Signaling Technology, Danvers, MA, USA) and mouse antibody against β‐actin (1:1000; cat. no. 981–28,721; Wako, Osaka, Japan). Horseradish peroxidase‐linked goat anti‐rabbit IgG secondary antibody (1:4000; cat. no. sc‐2004; Santa Cruz Biotechnology, Dallas, TX, USA) and horseradish peroxidase‐linked anti‐mouse IgG secondary antibody (1:2000; cat. no. 7076S; Cell Signaling Technology, Danvers, MA, USA) were used as secondary antibodies. Protein bands were quantified with ImageJ/Fiji software (version 2.1.0/1.53f; National Institutes of Health).

### 2.9 Small interfering RNA (siRNA) transfection

Colo320DM cells were plated in six‐well plates at 4.5 × 10^5^/well and transfected with 20 nmol/L siRNA targeting human CCR8 (5′‐CCCUAAAGGUGAGGACGAU‐3′) with the use of Lipofectamine® 3000 (Invitrogen, Walham, MA, USA) according to the manufacturer's protocols. The cells were then incubated for 72 h at 37°C after transfection. Non‐silencing siRNA (Ns siRNA) was used as the negative control.

### 2.10 Scratch test

The scratch test was implemented to determine cell motility. Colo320DM cells transfected with Ns siRNA or siRNA were seeded in 12‐well plates at 2.5 × 10^6^/well. When cells reached full confluency, scratches were made on the monolayer with a 10 μL pipette tip. After removing cell debris, the scratched monolayer was cultured with RPMI 1640 medium with or without rCCL1 for 60 h at 37°C. Cell motility was evaluated by using IncuCyte ZOOM (Sartorius, Göttingen, Germany). Similarly, Colo320DM cells pretreated with MK‐2206 dihydrochloride were examined to assess AKT signalling pathway‐induced cell motility, and seeded in 12‐well plates at 2.5 × 10^6^/well; the scratch test was then carried out under the same conditions.

### 2.11 Statistical analysis

Statistical analysis was carried out with IBM SPSS Statistics. The data are presented as the means ± standard deviation. *p* < 0.05 was considered statistically significant.

## 3 RESULTS

### 3.1 RNA sequencing of resected CRC and CRLM specimens suggests that CRLM is less enriched in CCL1

**FIGURE 1:**
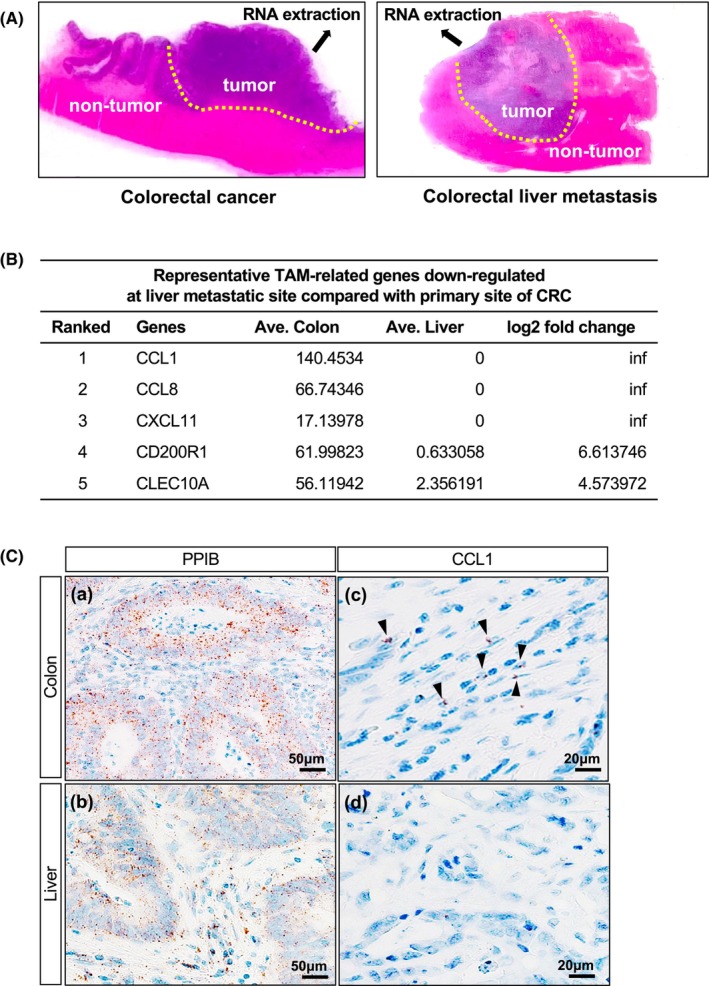
Total RNA was extracted and trimmed from formalin‐fixed, paraffin‐embedded (FFPE) sections of three cases of CRC and CRLM (A, dotted line) and subjected to RNA sequencing. The top five genes related to the decreased expression of TAMs in CRLM compared with that in CRC are listed as targeted genes. The data are normalized by TPM (B). RNA scope® assay displays CCL1 mRNA in the FFPE tissues from CRC and CRLM. The housekeeping gene PPIB stained positive in CRC and CRLM (C [a, b]). Little CCL1 mRNA expression is detected in the stroma of CRC (C [c], black arrowheads), while none is detected in that of CRLM (C [d]).

As shown in Figure 1A, total RNA (5‐μm) was extracted and trimmed from the paraffin sections of CRC and CRLM tumour tissues. The RNA integrity number (RIN) of samples was approximately 2.5. Although RNA degradation is observed in FFPE tissues as compared with that in fresh frozen tissues, the appropriate RNA extraction method according to the RIN value enables genetic analysis. Consequently, ribosomal RNA (rRNA)‐depleted RNA sequencing was carried out. On average, over 6 million (M) reads were generated per sample. After read trimming and filtering, genome mapping rates were 70% on average. Finally, down‐regulated genes at liver metastatic sites related to TAMs were selected as the top of five candidate genes (Figure 1B). The CCL1 gene was selected for further study by considering the following: Among the five top candidate genes, CCL1 gene expression has the largest gap between CRC primary sites and liver metastatic sites; previous studies have demonstrated that CCL1 progresses clinically and biologically in several solid tumours,^14^, ^15^, ^16^, ^17^ which might also support the mechanism of altered tumour activity in our study.

### 3.2 In situ detection of CCL1 mRNA in FFPE tissues

To confirm RNA sequencing results, the expression of CCL1 mRNA was detected by using RNA scope® in situ hybridization of CRC and CRLM FFPE tissues. As shown in Figure 1C [a, b], the positive control probe PPIB was hybridized evenly in all the tumour cells as well as in the stroma of CRC and CRLM. Target probe CCL1 was hybridized mostly in the stroma of CRC (Figure 1C [c]), whereas little or no expression of CCL1 was detected in the stroma and tumour cells of CRLM (Figure 1C [d]).

### 3.3 Identification of CCL1 and CCR8 in CRC and CRLM tissues

**FIGURE 2:**
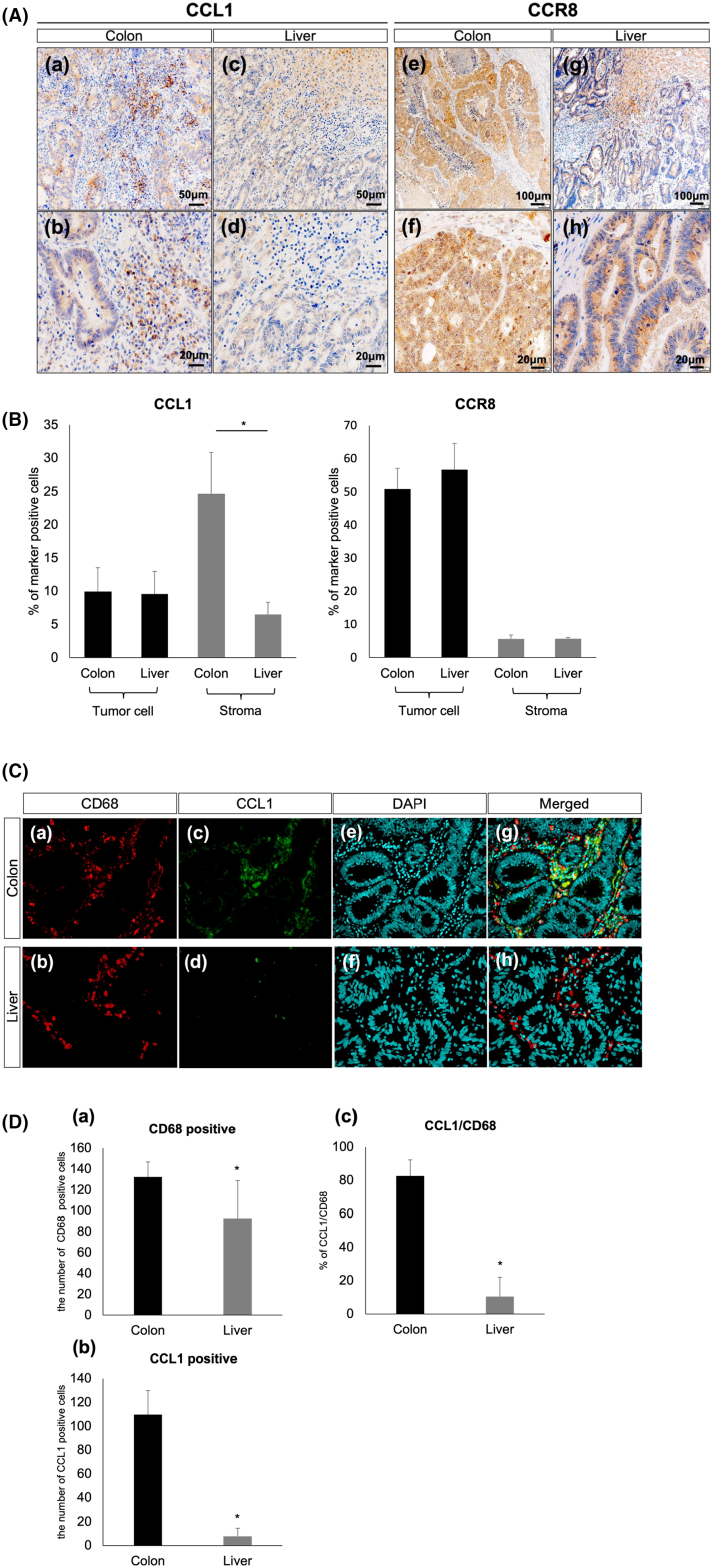
Immunohistochemical expression of CCL1 and CCR8 in 16 cases of CRC and CRLM (A) and quantification by the manual‐visual counting method (B). CCL1‐positive cells are accumulated mainly in the stroma of CRC (A [a,b]), while few low‐intensity CCL1‐positive cells are observed in the stroma of CRLM (A [c,d]). A significant difference is observed in the quantity of CCL‐1 positive cells between CRC and CRLM. A significant difference is observed in the distribution of CCR8‐positive cells in CRC and CRLM (A [e–h]). Double immunofluorescence staining of CCL1 (green) and CD68 (red) with DAPI (blue) in CRC and CRLM (C). CD68 and CCL1 immunoreactivity is observed more in CRC than in CRLM (C [a–d], D [a, b]). CCL1 immunoreactivity is colocalized mostly with CD68 in CRC (C [g]), but rarely in CRLM (C [h]). The percentage of CCL1 in CD68 positive cells (CCL1/CD68) was significantly higher in CRC than in CRLM (D [c]). *p < 0.05.

To identify the expression of CCL1 and CCR8 in CRC and CRLM, sections of FFPE tissues obtained at surgery from the 16 subjects were analysed by immunohistochemistry (IHC). The number of CCL1‐positive cells located in the stroma of CRC was significantly larger than that in CRLM (24.7 ± 6.2% vs. 6.5 ± 1.8%; *p* < 0.05). On the other hand, CCL1 in the tumour cells of both CRC and CRLM stained weakly (9.9 ± 3.6% vs. 9.6 ± 3.4%; *p* = 0.54), (Figure 2A [a–d], Figure 2B). The number of CCR8‐positive cells in the tumour cells was significantly higher than in the stroma of both CRC and CRLM (50.9 ± 6.3%, 56.8 ± 7.8% vs. 5.6 ± 1.1%, 5.7 ± 0.4%; *p* < 0.05) (Figure 2A [e–h], Figure 2B). No significant staining was observed in the negative controls. The CCL1 and CCR8 positive cells were measured through the analysis of a percentage of total stromal cells on five high‐power fields by two independent observers.

### 3.4 The altered character of TAMs in CRC and CRLM might associate with tumour progression

CD68 was positive in macrophages of the stroma of CRC and CRLM (132.2 ± 14.4 vs. 92.5 ± 36.2; *p* < 0.05) (Figure 2C [a,b], Figure 2D [a]). CCL1 immunoreactivity was mostly confined to CD68 positive macrophages in CRC compared with those in CRLM (109.6 ± 20.1 vs. 7.8 ± 6.8; *p* < 0.05) (Figure 2C [c,d], Figure 2D [b]). The percentage of CCL1 in CD68 positive cells was significantly higher in CRC than in CRLM (82.6 ± 9.6 vs. 10.4 ± 11.6; *p* < 0.05) (Figure 2C [g,h], Figure 2D [c]). Quantification of images was done on 10 high‐power fields by two independent observers.

### 3.5 Selection of CCR8‐positive cell line

**FIGURE 3:**
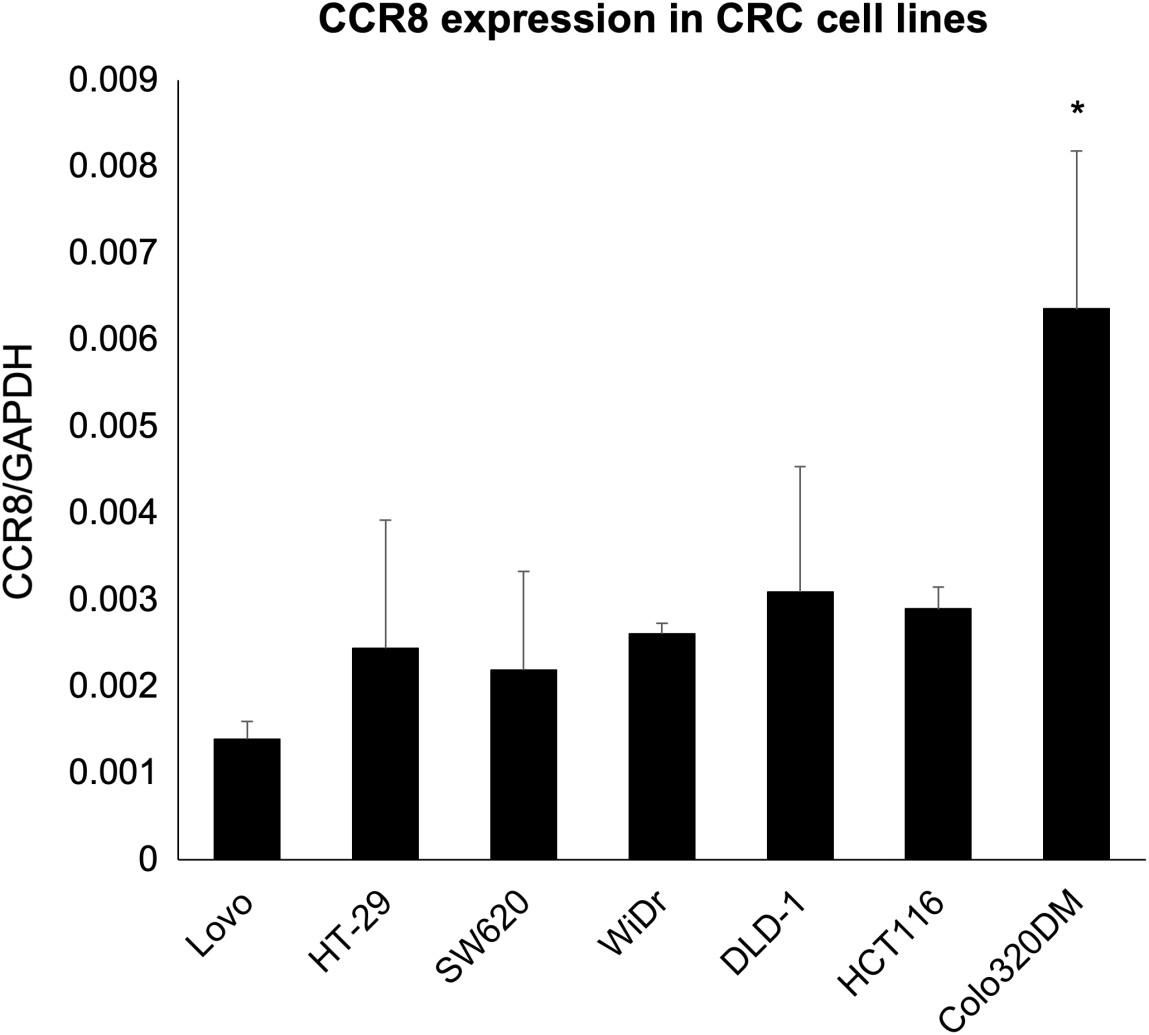
CCR8 mRNA expression in CRC cell lines (Lovo, HT‐29, SW620, WiDr, DLD‐1, HCT116 and Colo320DM) measured by RT‐qPCR with the SYBR Green technique. Expression of CCR8 mRNA is higher in Colo320DM cells than in the other cells. The data are normalized to internal control GAPDH. *p < 0.05.

In elucidating the molecular mechanisms of the CCL1‐CCR8 axis in CRC, the CRC cell lines were used to observe ‘dynamic property’, such as cell motility. By screening CCR8‐positive cell lines shown in Figure 3, the expression of CCR8 mRNA was significantly high only in the Colo320DM cell line that was used to evaluate the CCL1‐CCR8 axis.

### 3.6 CCL1 possibly activates AKT signalling pathway via CCR8 in Colo320DM cells

**FIGURE 4:**
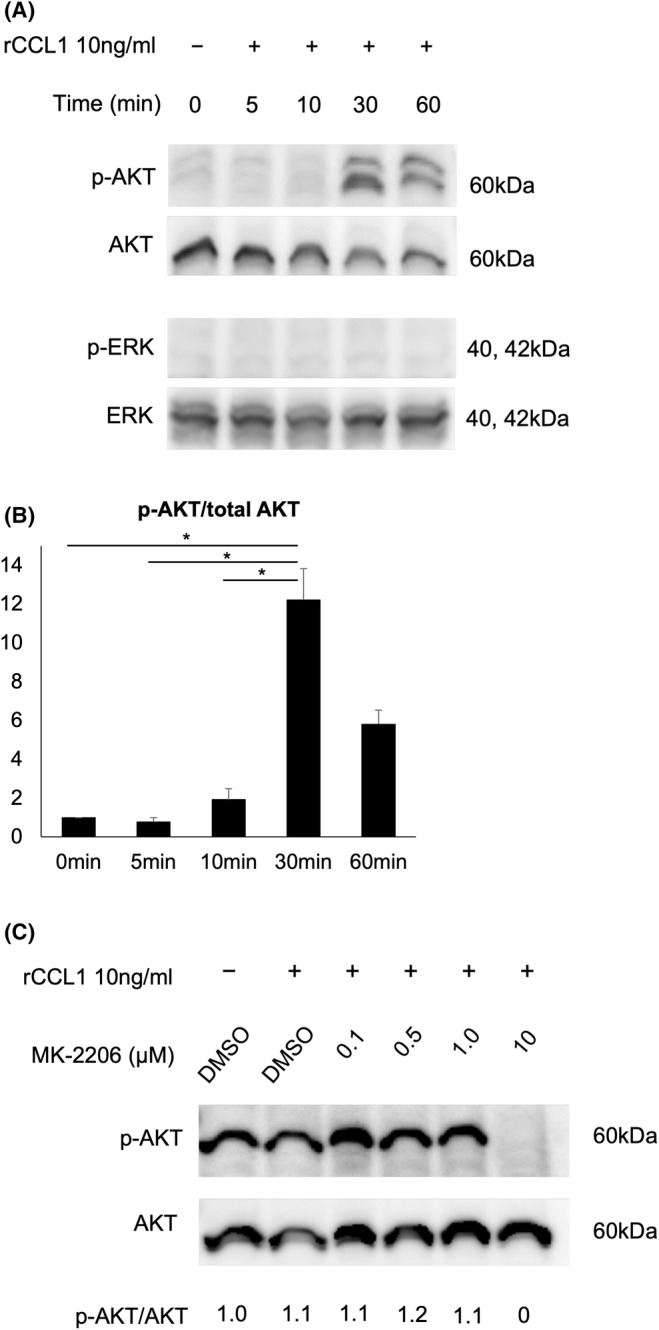
Western blotting detected the levels of phosphorylated AKT (p‐AKT), total AKT, phosphorylated ERK (p‐ERK) and total ERK in Colo320DM cells treated with rCCL1 (10 ng/mL) for 0, 5, 10, 30, 60 min (A). p‐AKT/AKT expression in Colo320DM cells peaked at 30 min after rCCL1 treatment, whereas no expression of p‐ERK/ERK after rCCL1 treatment is observed (B). Inhibition of AKT phosphorylation in Colo320DM cells by treatment with 0.1, 0.5, 1.0 and 10 μM MK‐2206 dihydrochloride for 24 h is detected by Western blotting. Reduced p‐AKT level is observed by treatment with 10 μM MK‐2206 dihydrochloride (C). *p < 0.05.

Investigation of the CCL1‐induced signalling pathways in Colo320DM cells showed that phosphorylated AKT (p‐AKT) peaked at about 30 min upon rCCL1 stimulation and peaked out at about 60 min. On the other hand, total ERK protein remained constant, and phosphorylated ERK (p‐ERK) was not observed (Figure 4A,B). Subsequently, pretreatment of Colo320DM cells with MK‐2206 dihydrochloride, an AKT inhibitor, was carried out with 0.1, 0.5, 1.0 and 10 μM for 24 h, and AKT phosphorylation was examined at 30 min after rCCL1 stimulation. As shown in Figure 4C, AKT phosphorylation was almost inhibited with 10 μM, while total AKT protein remained constant.

### 3.7 CCL1‐CCR8 axis regulates MMP‐2 expression in Colo320DM cells

**FIGURE 5:**
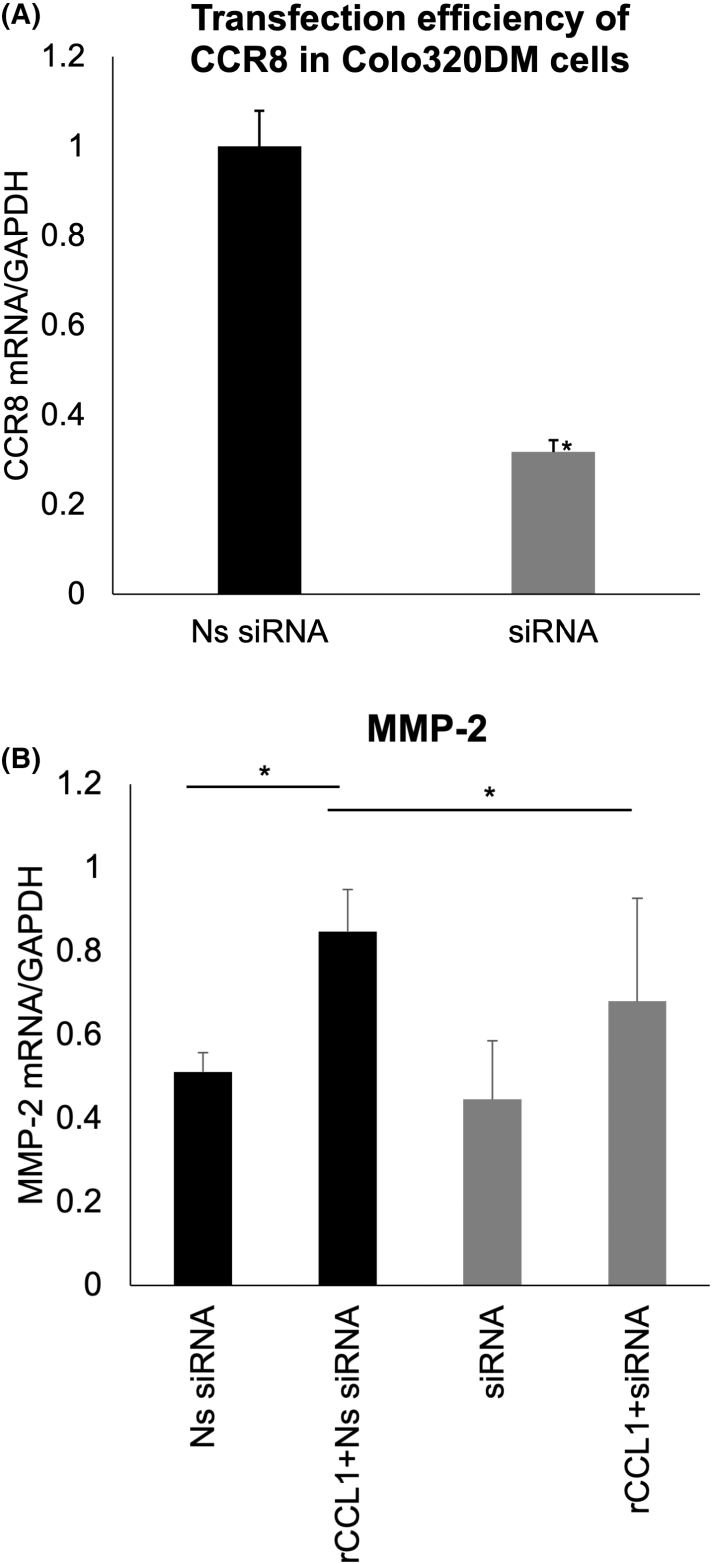
mRNA expression levels of CCR8, MMP‐2, MMP‐9, CDH1 and VEGFA in Colo320DM cells transfected with Ns siRNA or siRNA measured by RT‐qPCR. Transfection efficiency of CCR8 in Colo320DM cells measured by RT‐qPCR. Ns siRNA and siRNA were transfected into Colo320DM cells using Lipofectamine® 3000. Relative expression of CCR8 mRNA is significantly knocked down in Colo320DM cells transfected with siRNA (A). Expression of MMP‐2 in Colo320DM transfected with Ns siRNA plus rCCL1 (10 ng/mL) is significantly higher than transfection with siRNA plus rCCL1 (B). The data are normalized to internal control GAPDH. *p < 0.05.

To investigate whether the CCL1‐CCR8 axis is involved in tumour invasion and metastasis through epithelial‐mesenchymal transition (EMT) and tumour angiogenesis in Colo320DM cells, RT‐qPCR with siRNA treatment was conducted to evaluate the expression of CDH1, MMP‐2, MMP‐9 and VEGFA mRNA. CCR8 expression in Colo320DM cells transfected with siRNA decreased by about 70% in cells transfected with Ns siRNA as the negative control (Figure 5A). On the other hand, MMP‐2 mRNA expression in Colo320DM cells transfected with Ns siRNA increased significantly upon treatment with rCCL1 (10 ng/mL), while no change in MMP‐2 mRNA expression was observed in cells transfected with only siRNA (Figure 5B). Similarly, VEGFA mRNA expression in Colo320DM cells transfected with siRNA increased slightly after treatment with rCCL1, with no statistically significant difference (Figure S1A, *p* = 0.19). Also, no significant difference was observed in either MMP‐9 or CDH1 (Figure S1B,C, *p* = 0.90, *p* = 0.92).

### 3.8 CCL1‐CCR8 axis promotes motility of Colo320DM cells by the AKT signalling pathway

**FIGURE 6:**
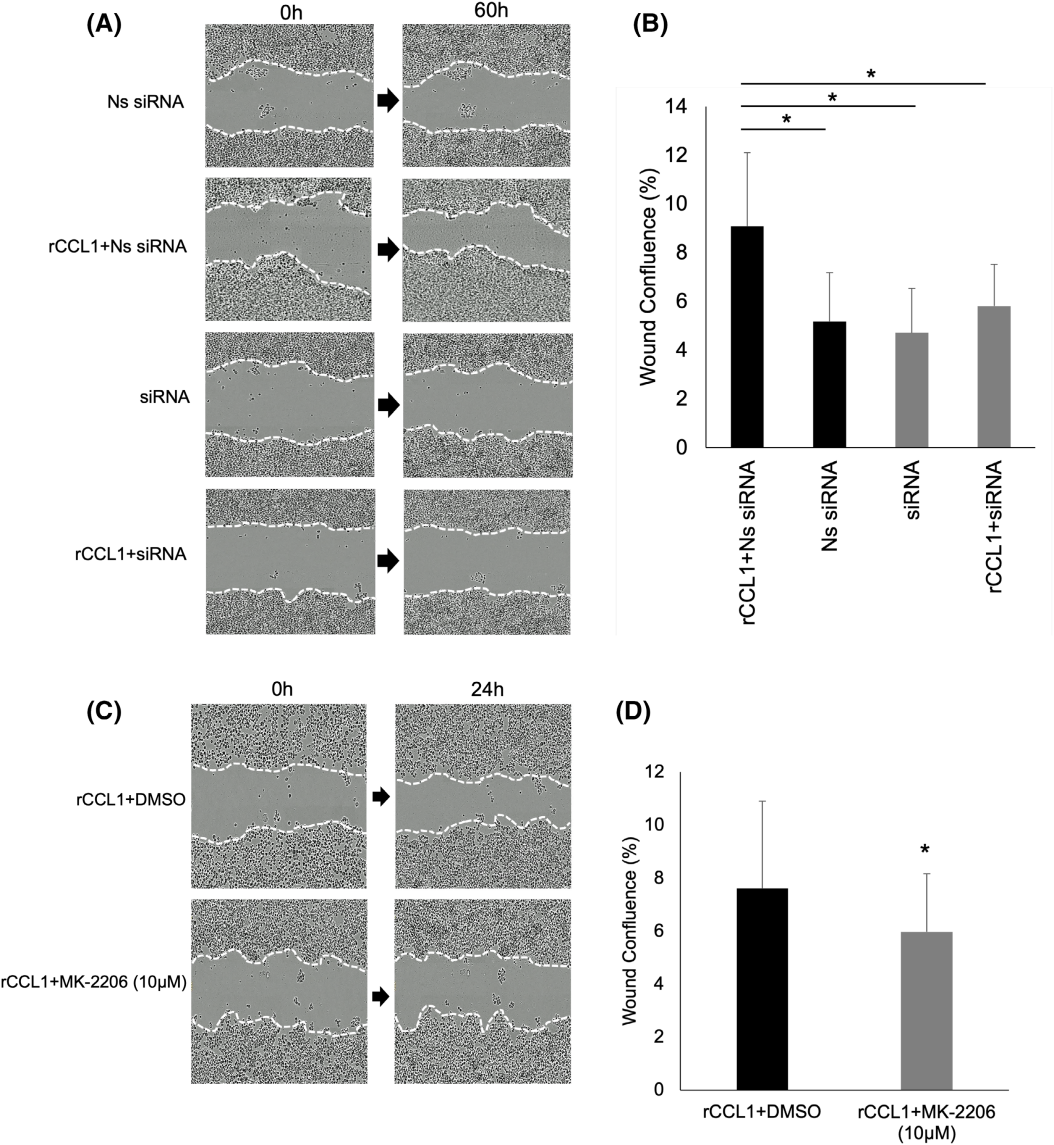
The scratch test demonstrates a significant difference of cell motility between Colo320DM cells transfected with rCCL1 and Ns siRNA than with rCCL1 and siRNA. Representative time‐lapse images of scratch test (A). Each image was taken immediately and 60 h after the scratch assay. Quantitative analysis was carried out with IncuCyte ZOOM (B). Views of the scratch test after treatment with DMSO and MK‐2206 dihydrochloride (C). 10 μM MK‐2206 dihydrochloride markedly reduced rCCL1‐dependent cell motility in Colo320DM cells (D). *p < 0.05.

To evaluate the effect of CCL1 on cell motility of CRC, the scratch test conducted on Colo320DM cells transfected with Ns siRNA as a negative control showed significant cell motility upon treatment with rCCL1 (10 ng/mL), whereas CCR8 knockdown groups transfected with siRNA showed low cell motility upon treatment with rCCL1 (Figure 6A,B). Next, to determine whether the AKT signalling pathway is involved in rCCL1‐enhanced cell motility, the scratch test was conducted in the presence or absence of an AKT inhibitor. As shown in Figure 6C,D, pretreatment with 10 μM MK‐2206 dihydrochloride for 24 h significantly decreased cell motility of Colo320DM cells. Changes in wound confluence (%) over time and the time‐lapsed video are shown in Figure S2A–D.

## 4 DISCUSSION

Since surgical resection of liver metastatic sites improves CRC patient prognosis,^3^, ^4^, ^5^ the clinical realization that the potential for tumour activity decreasing at liver metastatic sites compared with that at primary sites was emerged. Moreover, among factors that constitute TME, TAMs have been described as one that supports tumour progression in CRC.^9^, ^10^ Therefore, assuming that alterations in the character of TAMs at liver metastatic sites decelerates CRC progression, comprehensive genetic analysis by RNA sequencing was implemented to isolate the differentially expressed genes in TAMs at metastatic sites compared with those at primary sites. In the present study, comprehensive RNA sequencing data were obtained from three independent pairs of FFPE samples as opposed to data from fresh frozen tissues (Figure 1A). RNA scope® in situ hybridization also validated the difference between primary and metastatic sites, confirming the RNA sequencing data (Figure 1C); therefore, CCL1 which has the largest gap between CRC primary sites and liver metastatic sites was selected for further study.

CCR8 is the sole receptor for CCL1, and the CCL1‐CCR8 axis promotes tumour activity in various malignancies.^14^, ^15^, ^16^, ^17^ To date, the difference between CCL1 expression at CRC primary and metastatic sites has not been elucidated.

To screen and validate the expression of CCL1 and CCR8, IHC analysis was carried out on CRC and CRLM tissues. CCL1‐positive cells were located mainly in the stroma of CRC and CRLM, with the number in CRC being higher in CRLM (Figure 2A [a–d]). On the other hand, CCR8 was constantly observed among tumour cells at both primary and metastatic sites (Figure 2A [e–h]).

In solid tumours, CCL1 is produced by TME‐composed cells such as TAMs, CAFs and regulatory T cells (Treg), and acts through CCR8 expressed in tumour cells, vascular endothelial cells and Treg.^18^ To confirm the difference in the characteristics of TAMs in CRC and CRLM, double immunofluorescence staining of CCL1 and CD68, as pan macrophage markers, showed CD68 as positive in macrophages and stroma of CRC and CRLM (Figure 2C [a,b], Figure 2D [a]), and CCL1 positive cells were almost confined to CD68 positive macrophages in CRC compared with those in CRLM (Figure 2C [c,d], Figure 2D [b]). The percentage of CCL1 in CD68 positive cells (CCL1/CD68) was also significantly higher in CRC than in CRLM (Figure 2C [g,h], Figure 2D [c]). In other words, CD68 positive cells at the CRC primary site were largely CCL1‐positive macrophages, whereas those at the CRC metastatic site were largely CCL1‐negative macrophages. Thus, CCL1 plays a major role in altering the CCL1‐CCR8 axis in CRC and CRLM. Within CRLM, TAMs (differentiated from monocyte/macrophages recruited to the tumour site or resident liver macrophages, known as Kupffer cells) are mixed; they then switch their functional phenotypes, M1‐like or M2‐like macrophages, flexibly according to several signals from TME.^19^ Importantly, the difference of CCL1 expression level in CD68 positive cells at the primary site and in the liver may be caused by the predominance of Kupffer cells in the liver. Chemokine profiles of TAMs from different origins are likely distinct. Since M2 macrophages have three other phenotypes (M2a, M2b or M2c) and M2b polarization is characterized by selective production of CCL1,^20^ we assume that TAMs in CRC might differentiate to M2b and those in CRLM to the two others. In this study, pan macrophage marker CD68 was used for investigating TAMs since their polarization is inconstant; however, distinguishing two TAMs, monocyte/macrophages or resident macrophages, solely through the CD68 marker is complicated. We must also admit that there are technical limitations to examining the detailed characteristics of TAMs in paraffin sections.

With the use of human CRC cell lines, in vitro experiments were conducted to reveal the role of the CCL1‐CCR8 axis. In the present study, CCR8 expression levels were through RT‐qPCR screened also in several human colon carcinoma cell lines (Figure 3). Immunohistochemical examination revealed that while CCR8 was positive in most surgically resected tissues at primary and metastatic sites, except in Colo320DM, CCR8 expression level was low or absent in most cell lines tested. Changes in the original characters due to the process of repeated passages for establishing cell lines may have affected CCR8 expression. Colo320DM cells were selected to test the functionality of CCR8 through Western blotting for the evaluation of signalling pathways, RT‐qPCR for the evaluation of EMT induction, and the scratch assay for the evaluation of migration and invasion.

To further elucidate the signalling pathway mediated by the CCL1‐CCR8 axis, Western blotting was done to determine whether AKT and ERK signalling pathways are activated by CCL1 in Colo320DM cells (Figure 4A,B). Consequently, CCL1 might be involved mainly in tumour progression through the AKT signalling pathway in Colo320DM cells. A study on CCL1 signalling pathways has demonstrated that CCL1 derived from TAMs activates the AKT/PRAS40/mTOR signalling pathway through CCR8 in oesophageal squamous cell carcinoma (ESCC).^17^ In the present study, however, the activation of AKT upon rCCL1 stimulation was observed as a later event when compared with observations in a previous report.^17^ Therefore, we speculate that the CCL1‐CCR8 axis activates AKT signal secondarily through an upstream factor of AKT signalling pathways, such as the phosphatidylinositol‐3 kinase (PI3K). On the other hand, in adult T‐cell leukaemia, antiapoptotic activity due to the activation of the ERK/MAPK signalling pathway by the CCL1‐CCR8 axis induces chemoresistance to anticancer drugs.^21^ Another study has revealed that double minute chromosomes (DM)‐containing tumour cells such as Colo320DM typically require the phosphorylation of ERK for the production or maintenance of DM cells^22^; however, the activation of p‐ERK upon rCCL1 stimulation was not observed in the present study. Thus, our findings suggest that CCL1 may suppress the ERK signalling pathway in Colo320DM cells.

EMT plays the important role of metastasis in various tumours involving CRC.^23^, ^24^, ^25^, ^26^, ^27^ Tumour cells induced by EMT‐related signalling pathways such as WNT/β‐catenin and TGF‐β lose their epithelial characteristics because of the downregulation of CDH1 and the gain of mesenchymal properties such as increased migration activity. Mesenchymal cells that gain migration activity are capable of migrating to lymph nodes and vessels through the extracellular matrix attributed to matrix metalloproteinase such as MMP‐2 and MMP‐9. VEGF is also one of the factors promoting EMT.^28^ In the present study, the change in CDH1, MMP‐2, MMP‐9 and VEGFA levels after their treatment with rCCL1 was assessed by RT‐qPCR to identify whether CCL1 induces EMT in Colo320DM cells. While the increased value per se was nominal, the increase in MMP‐2 levels in Colo320DM cells treated with rCCL1 was statistically significant (Figure 5B). These data suggested that CCL1 might also promote EMT through CCR8 in CRC. The MMP family is associated with tumour progression, metastasis and angiogenesis of CRC, and the overexpression of MMP‐2 and MMP‐9 has been known as a sign of poor prognostic factors.^29^ Colo320DM cells lack the characteristics of forming dense cell colonies and are easily detached from culture plates due to few microvilli or desmosomes.^30^, ^31^ Thus, Colo320DM cells which have originally lost epithelial characteristics could cause low expression of EMT‐related factors other than MMP‐2.

Cell motility of CRC through the CCL1‐CCR8 axis was measured with the scratch test. These data suggest that the CCL1‐CCR8 axis promotes cell motility by the AKT signalling pathway in Colo320DM cells. On the other hand, as mentioned above, Colo320DM cells originally lose epithelial characteristics, and therefore, the evaluation of cell motility through EMT has been excluded from this study.

Pulmonary metastasis is the second most common metastatic site of CRC.^25^ The 5‐year OS rate of patients after surgical treatment for pulmonary metastasis of CRC is 30%–68%, and has recently been considered comparable to the outcome for liver metastasis after hepatectomy.^32^, ^33^ The efficacy of the surgical treatment of patients with synchronous hepatic and pulmonary metastasis of CRC has also been described.^34^ Based on our study, the expression of CCL1 might decrease at pulmonary and liver metastatic sites; however, verification is pending. Further studies involving a larger number of liver and pulmonary metastatic sites are needed.

Tumour location (left‐sided vs. right‐sided) and developmental pathways in CRC (‘de novo’ carcinogenesis, adenoma‐carcinoma sequence or serrated‐neoplasia sequence) have been known to influence tumour activity and prognosis.^35^, ^36^ In the present study, 13 of the 16 subjects demonstrated left‐side colon cancer, not including those of young‐onset CRC. Therefore, all cases were assumed to have developed through the conventional adenoma‐carcinoma sequence, since nearly 70% of CRC cases develop through this sequence. Further studies are warranted for identifying the association between CCL1 expression levels and the prognosis of CRC in terms of tumour location and developmental pathways such as ‘de novo’ carcinogenesis and serrated‐neoplasia sequences.

In conclusion, the present study revealed that TAMs at CRC primary and metastatic sites have different characteristics, suggesting that decreased CCL1 expression of TAMs at liver metastatic sites partly explains the unique slow tumour progression of CRC, providing for a grace period for radical resection of metastatic lesions.

## AUTHOR CONTRIBUTIONS

**Miku Iwata:** Data curation (lead); formal analysis (lead); investigation (lead); validation (lead); visualization (lead); writing – original draft (lead). **Sohei Kitazawa:** Conceptualization (lead); funding acquisition (lead); project administration (lead); resources (lead); supervision (lead); writing – original draft (lead); writing – review and editing (lead). **Ryuma Haraguchi:** Formal analysis (supporting); investigation (supporting); methodology (supporting); supervision (supporting); validation (supporting); writing – review and editing (supporting). **Riko Kitazawa:** Conceptualization (lead); funding acquisition (lead); resources (lead); supervision (lead); writing – review and editing (supporting). **Chihiro Ito:** Investigation (supporting); writing – review and editing (supporting). **Kohei Ogawa:** Supervision (supporting); writing – review and editing (supporting). **Yasutsugu Takada:** Funding acquisition (supporting); supervision (supporting); writing – review and editing (supporting).

## CONFLICT OF INTEREST STATEMENT

The authors declare no conflicts of interest.

## Supporting information


Figure S1.

Table S1.

Figure S2.

## Data Availability

The data supporting the findings of this study are available from the corresponding author upon reasonable request. Sequencing data are available at the DNA Data Bank of Japan (DDBJ) Sequence Read Archive (DRA) under accession number DRA016565.
