# Supplementary material for: Reduced chemokine C‐C motif ligand 1 expression may negatively regulate colorectal cancer progression at liver metastatic sites
Source: J Cell Mol Med. 2024 Mar 20;28(7):e18193. doi: 10.1111/jcmm.18193 (PMC10952021; doi:10.1111/jcmm.18193)
Supplement: Supplementary file 1 — Figure S1. [file JCMM-28-e18193-s001.pdf]

Reduced Chemokine C-C motif ligand 1 (CCL1) expression may negatively regulate colorectal cancer progression at liver metastatic site

Miku Iwata, Ryuma Haraguchi, Riko Kitazawa, Chihiro Ito, Kohei Ogawa, Yasutsugu Takada and Sohei Kitazawa

- ✓ **Supplementary Figure 1 (A-C)**
- ✓ **Supplementary Figure 2 (A, C)**
- ✓ **Supplementary Figure 2 (B, D) are Videos, uploaded as separate media file**

Supplementary Figure 1

(A)

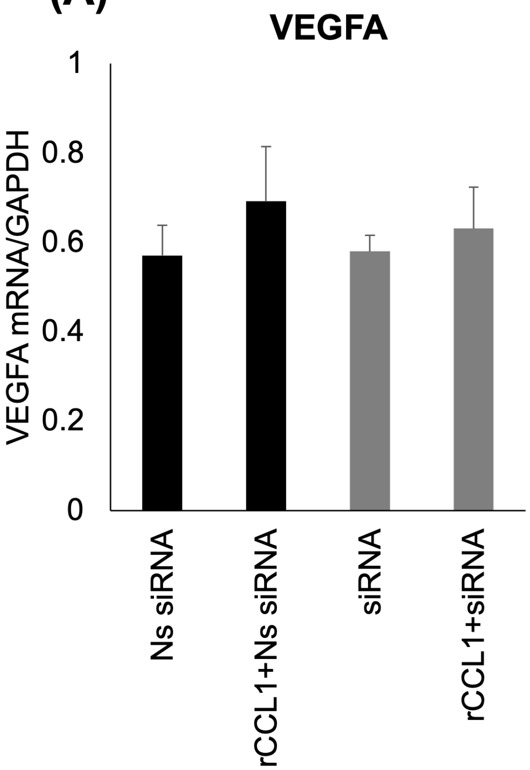

(B)

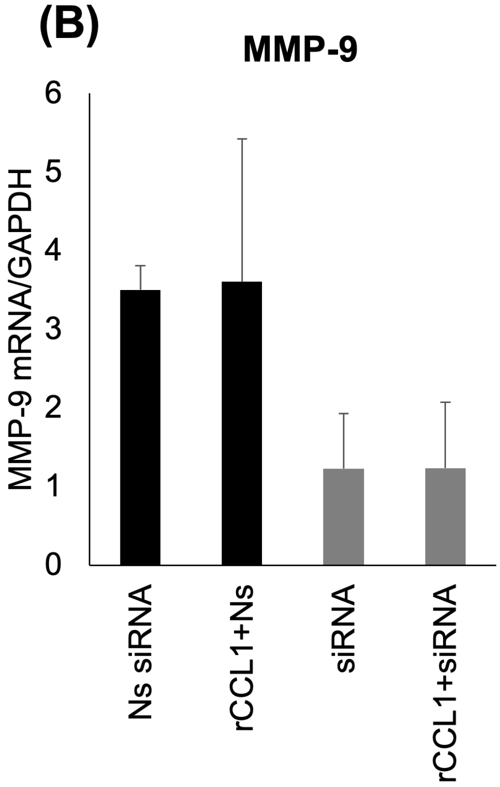

(C)

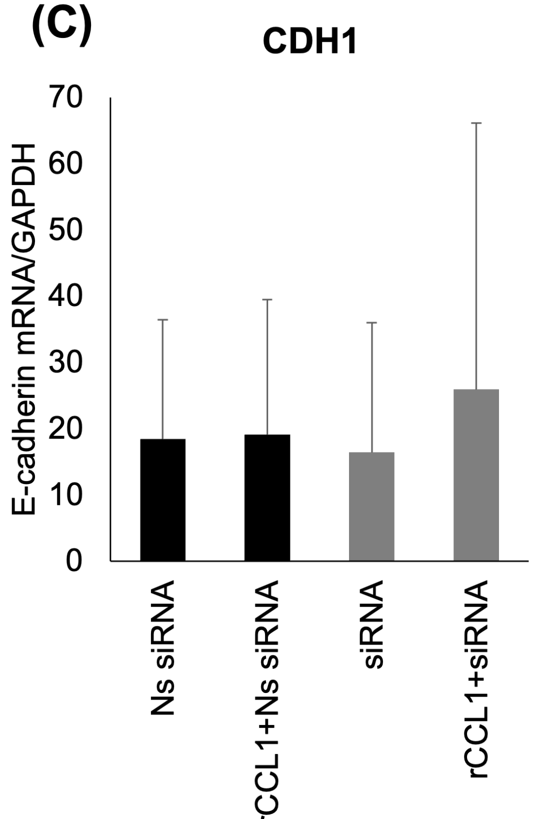

Expression of VEGFA in Colo320DM cells transfected with Ns siRNA added to rCCL1 (10ng/ml) were mildly increased compared with no rCCL1, however, there was no significant difference (A, p=0.19). MMP-9 and CDH1 were showed no effects of rCCL1 (B, C, p=0.92, p=0.90). The data were normalized to internal control GAPDH. *P<0.05.

Supplementary Figure 2

(A)

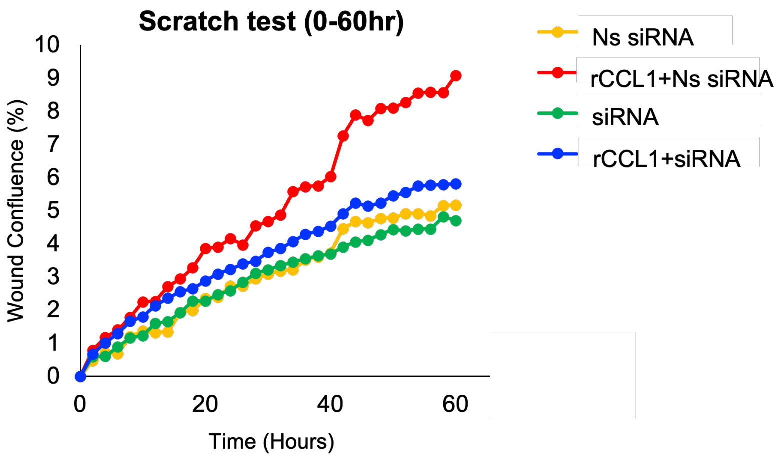

(C)

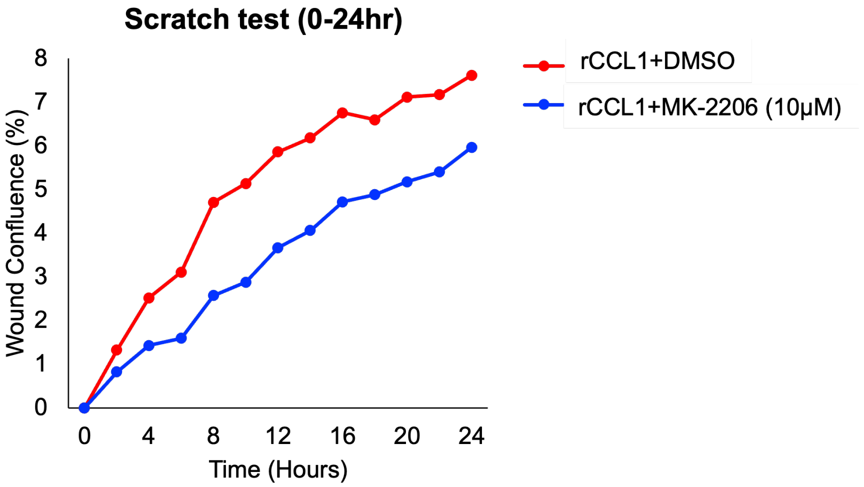

Supplementary Figure 2 (B, D) are Videos, uploaded as separate media file

Changes of wound confluence (%) in Colo320DM cells over the time (A). The time-lapsed video (B). Changes of wound confluence (%) in Colo320DM cells after 10μM MK-2206 dihydrochloride pretreatment (C). The time-lapsed video (D).
