# Supplementary material for: Reduced chemokine C‐C motif ligand 1 expression may negatively regulate colorectal cancer progression at liver metastatic sites
Source: J Cell Mol Med. 2024 Mar 20;28(7):e18193. doi: 10.1111/jcmm.18193 (PMC10952021; doi:10.1111/jcmm.18193)
Supplement: Supplementary file 2 — Table S1. [file JCMM-28-e18193-s003.pdf]

Reduced Chemokine C-C motif ligand 1 (CCL1) expression may negatively regulate colorectal cancer progression at liver metastatic site

Miku Iwata, Ryuma Haraguchi, Riko Kitazawa, Chihiro Ito, Kohei Ogawa, Yasutsugu Takada and Sohei Kitazawa

- ✓ **Supplementary Table 1**

Supplementary Table 1

Sample ID	Total number of sequenced reads	Total number of uniquely mapped reads	RNA integrity number (RIN)	Ratio of all reads aligned to rRNA regions to total uniquely mapped reads (rRNA rate)	Ratio of exon-mapped reads to total uniquely mapped reads (Expression Profile Efficiency)	Total number of detected transcripts with reads ≥1
H2101094_liver_tumor	5460000	3754350.6	2.3	0.893	0.37	11448
H1906298_colon_tumor	5060000	2568091.68	2.5	0.798	0.08	9724
H1902323_liver_tumor	8160000	3048510.72	2.5	0.697	0.08	7871
H1901060_colon_tumor	5360000	2034446.96	2.5	0.679	0.03	7251
H2004321_liver_tumor	4720000	3900768.48	2.4	0.937	0.27	11960
H1903794_colon_tumor	5660000	3853956.26	2.3	0.8365	0.19	11218

The summary of the sequencing coverage and quality statistics of each sample. The hg38 reference genome was used.
